# Hypothermia but not NMDA receptor antagonism protects against stroke induced by distal middle cerebral arterial occlusion in mice

**DOI:** 10.1371/journal.pone.0229499

**Published:** 2020-03-03

**Authors:** Che-Wei Liu, Kate Hsiurong Liao, Hsin Tseng, Ching Mei Wu, Hsiao-Yun Chen, Ted Weita Lai

**Affiliations:** 1 Graduate Institute of Biomedical Sciences, China Medical University, Taichung, Taiwan; 2 School of Medicine, China Medical University, Taichung, Taiwan; 3 Graduate Institute of Clinical Medical Science, China Medical University, Taichung, Taiwan; 4 Department of Anesthesiology, China Medical University Hospital, Taichung, Taiwan; 5 Drug Development Center, China Medical University, Taichung, Taiwan; 6 Translational Medicine Research Center, China Medical University Hospital, Taichung, Taiwan; Massachusetts General Hospital/Harvard Medical School, UNITED STATES

## Abstract

Excitotoxicity mediated by the N-methyl-D-aspartate receptor (NMDAR) is believed to be a primary mechanism of neuronal injury following stroke. Thus, many drugs and therapeutic peptides were developed to inhibit either the NMDAR at the cell surface or its downstream intracellular death-signaling cascades. Nevertheless, the majority of focal ischemia studies concerning NMDAR antagonism were performed using the intraluminal suture-induced middle cerebral arterial occlusion (MCAO) model, which produces a large cortical and subcortical infarct leading to hypothalamic damage and fever in experimental animals. Here, we investigated whether NMDAR antagonism by drugs and therapeutic peptides was neuroprotective in a mouse model of distal MCAO (dMCAO), which produces a small cortical infarct sparing the hypothalamus and other subcortical structures. For establishment of this model, mice were subjected to dMCAO under normothermic conditions or body-temperature manipulations, and in the former case, their brains were collected at 3–72 h post-ischemia to follow the infarct development. These mice developed cortical infarction 6 h post-ischemia, which matured by 24–48 h post-ischemia. Consistent with the hypothesis that the delayed infarction in this model can be alleviated by neuroprotective interventions, hypothermia strongly protected the mouse brain against cerebral infarction in this model. To evaluate the therapeutic efficacy of NMDAR antagonism in this model, we treated the mice with MK801, Tat-NR2B9c, and L-JNKI-1 at doses that were neuroprotective in the MCAO model, and 30 min later, they were subjected to 120 min of dMCAO either in the awake state or under anesthesia with normothermic controls. Nevertheless, NMDAR antagonism, despite exerting pharmacological effects on mouse behavior, repeatedly failed to show neuroprotection against cerebral infarction in this model. The lack of efficacy of these treatments is reminiscent of the recurrent failure of NMDAR antagonism in clinical trials. While our data do not exclude the possibility that these treatments could be effective at a different dose or treatment regimen, they emphasize the need to test drug efficacy in different stroke models before optimal doses and treatment regimens can be selected for clinical trials.

## Introduction

Although N-methyl-D-aspartate receptor (NMDAR) antagonists effectively protect the brain against ischemic stroke damage in animals, these drugs have not shown beneficial effects in stroke patients in clinical trials [1]. The explanation for this clinical failure includes unanticipated adverse effects and a lack of therapeutic efficacy; hence, the suitability of the animal stroke models in which these drugs were initially tested for predicting clinical success has been questioned. Indeed, the neuroprotective efficacy of NMDAR antagonism can vary depending on the cerebral ischemia model used. For example, the prototypical NMDAR blocker MK801 (dizocilpine) has been shown to be neuroprotective against stroke (focal ischemia) in rats [2–7], mice [8], and cats [9, 10]; mild global ischemia in rats [11–13]; and hypoxia in neonatal rats [14–16] but failed to protect against neuronal injury caused by severe global ischemia in rats [17–19] and piglets [20]. The neuroprotective efficacy, or the lack thereof, is also dependent on the body temperature of the animals during the experiments. Although MK801 was initially thought to be neuroprotective against global ischemia in gerbils [21], further studies have shown that this drug produces marked hypothermia to achieve neuroprotection in gerbils and that it has little or no protection against global ischemia under normothermic conditions [22–24]. In comparison, MK801 had little or no effect on body temperature in adult and neonatal rats [15, 25, 26]. Nevertheless, the neuroprotective effect of MK801 was enhanced under hypothermic conditions in neonatal rats subjected to hypoxia [15, 26] and was abolished under spontaneous hyperthermia in adult rats subjected to focal ischemia [25, 27]. Therefore, the experimental model used and the body temperature of animals during stroke can dictate the therapeutic efficacy of neuroprotective drugs.

Most preclinical stroke studies, including all of the focal ischemia studies mentioned above, tested drug efficacy in experimental animals subjected to one of the middle cerebral arterial occlusion (MCAO) models; this method induces a large hemispheric infarct covering the cerebral cortex and many subcortical structures. These animals tend to develop pathophysiological and behavioral changes that could exacerbate the stroke outcome; as a result, the perceived treatment efficacy may not be due to neuroprotection *per se*. For example, the intraluminal suture-insertion MCAO model, which is by far the most popular of the MCAO models, damages the striatum to severely hinder mobility (including the ability to procure food) and damages the hypothalamus to cause fever that can further exacerbate neuronal injury [28, 29]. While the large infarct produced by the MCAO models can reflect the pathophysiology of some stroke patients, preclinical testing of neuroprotective drugs in additional models can provide further guidance on drug mechanisms of action and suitability for specific patient populations.

In the present study, we evaluated the neuroprotective efficacy of MK801 using a mouse model of transient dMCAO, which produces a small infarct confined to the cerebral cortex, sparing the striatum, hypothalamus, and other subcortical structures. Similar to that in the MCAO models [30], the cerebral infarction after dMCAO evolved progressively over the first 24 h after stroke, suggesting that the ischemic damage can be alleviated by neuroprotective strategies. Consistent with this hypothesis, hypothermia during the ischemic period dramatically reduced the infarct volume in these mice. Nevertheless, neither MK801, which inhibits NMDAR at the cell surface level, nor two therapeutic peptides, which inhibit the death-signaling cascades downstream of NMDAR, were neuroprotective.

## Materials and methods

### Animals

Male C57BL/6 mice (7–10 weeks in age and 21–30 g in weight) purchased from the National Laboratory Animal Center (Taipei, Taiwan) were used in this study. They were housed in large-diameter cages in groups of 10 per cage with standard rodent chow and water *ad libitum*. The room lighting was controlled under a 12:12 h light/dark cycle. The experimental protocols were carried out in accordance with the ARRIVE guidelines and the Institutional Guidelines of the China Medical University for the Care and Use of Experimental Animals (IGCMU-CUEA). All procedures were approved by the Institutional Animal Care and Use Committee (IACUC) of the China Medical University (Taichung, Taiwan) (Protocol No. 103-224-NH and No. CMUIACUC-2018-316).

### Cerebral ischemia

Continuous inhalation of air-carried isoflurane (1.5%–2.0%) was used for anesthesia during surgery, and subcutaneous bupivacaine (0.5%) was preemptively applied at surgical sites to minimize postoperative pain. The mice were subjected to transient dMCAO coupled with brief ipsilateral common carotid arterial (CCA) occlusion using the protocol of Chen *et al*. [31] as described previously [32]. In brief, a small craniotomy on the right temporal bone was performed to facilitate reversible ligation of one distal branch of the right middle cerebral artery with a 10–0 nylon suture (see **S1A and S1B Fig**); this procedure was coupled with temporary ligation of the right CCA using sewing cotton thread. After the vascular occlusions, the mice were allowed to recover from anesthesia in a heated cage (SY-667, Shinetech Co., Ltd.), where they remained awake for 120 min before re-anaesthetization to facilitate the removal of the ligations on the middle cerebral artery and CCA to allow complete reperfusion. In experiments where body temperature control was desired during the 120-min ischemic period, the mice were kept anesthetized by isoflurane while placed on a rectal probe-coupled heating pad with automatic temperature adjustments (TC-1000, CWE, Inc.). After reperfusion, the surgical wounds were closed using 6–0 nylon sutures, and the mice were allowed to recover from anesthesia in their home cages.

### Body temperature manipulation during ischemia

For normothermic (~37°C) or to hyperthermic conditions (39–40°C) during the 120-min ischemic period, mice were kept anesthetized by isoflurane (0.5% - 1.5%) while placed on a rectal probe-coupled automatically adjusted heating pad. For induction of hypothermia, the mice were anesthetized with 1% isoflurane during the 120-min ischemic period without the heating pad. In these experiments, a mouse rectal probe was securely taped to the mouse tail to avoid data fluctuation, and body temperature was monitored in a continuous manner.

### Drug administration and monitoring

MK801 (cat.# ab120027) was purchased from Abcam. Tat-NR2B9c (cat.# HY-P0117; peptide sequence: YGRKKRRQRRRKLSSIESDV) and L-JNKI-1 (cat.# HY-P0069A; peptide sequence: GRKKRRQRRRPPRPKRPTTLNLFPQVPRSQD) were purchased from MedChemExpress, and their purities were reported to be 98.22% and 95.50%, respectively, according to HPLC/LCMS analytical data from the manufacturer. For confirmation that MK801 [4 mg/kg, intraperitoneal (i.p.)] was efficiently delivered to the brain, locomotor behavior in a subset of the mice was continuously monitored and recorded in an open-field test chamber as described previously [33]. In addition, for determination of the potential effect of NMDAR antagonism on the body temperature of the mice, MK801 (4 mg/kg), Tat-NR2B9c (8 mg/kg), L-JNKI-1 (8 mg/kg), or vehicle (saline at 4 ml/kg) was injected (i.p.), at a dose equal to or greater than that previously found to be neuroprotective in the suture-insertion MCAO model [34, 35], in the 1% isoflurane-sedated mice, while the body temperature was recorded with a rectal probe. The sedation minimized animal mobility, which can cause data fluctuation during measurements. For determination of the neuroprotective efficacy of NMDAR antagonism, MK801 (4 mg/kg), Tat-NR2B9c (8 mg/kg), L-JNKI-1 (8 mg/kg), or vehicle (saline at 4 ml/kg) was injected (i.p.) 30 min prior to ischemia onset.

### Determination of the brain infarct volume

The mice were euthanized by an overdose of urethane (4 g/kg, i.p.) at 3, 6, 12, 24, 48 or 72 h after stroke, and coronal sections of the isolated brains were stained with 4% 2,3,5-triphenyltetrazolium chloride as previously described [36]. The infarct area per coronal section and the total infarct volume were quantified with the image analysis software ImageJ.

### Fluoro-Jade (FJ) staining

Neuronal death was histologically assessed by staining the brain slices with FJ B, as described previously [36]. In brief, the mice were euthanized and subjected to intracardiac perfusion with 4°C iced saline for 3 min. The motor cortex and hippocampus were then dissected and postfixed in 4% paraformaldehyde in PBS overnight. The brain sections were subsequently dehydrated overnight in 20%, 30%, and 40% sucrose (w/v) overnight until equilibration. The brain sections were then frozen in OCT mounting medium (#6769006, Thermo Scientific), and 25 μm coronal sections were cut and placed in 1x PBS. The sections were subsequently mounted on microscope slides and allowed to air-dry. Then, the sections were subsequently incubated in 100% ethanol for 3 min, 70% ethanol for 1 min, and distilled water for 1 min for 3 washes. The samples were oxidized by drenching in 0.06% KMnO_4_ for 15 min and then washed 3 times in distilled water for 1 min each. Next, the sections were stained in 0.0001% FJ B (AG310-30MG, Merck) in 0.1% acetic acid for 30 min. The slides were then washed 5 times in distilled water for 1 min each. After air-drying overnight at room temperature, the slides were mounted using Permount (Thermo Scientific). Digital images of FJ B-positive cells in the brain sections were collected on a laser confocal microscope (TCS SP8-X, Leica). Confocal images of a single optical plane (1 μm thick) were obtained at the appropriate excitation/emission wavelengths. Scanned images of the whole brain sections were obtained using a high-speed confocal system (Dragonfly 200, Andor) with a 488 mm FITC filter. The settings for confocal microscopy, laser attenuation, pinhole diameter, and photomultiplier sensitivity were determined by the microscope instructions and were consistently maintained in the experiments.

### Randomization and exclusion

All experiments involving cerebral ischemia were carried out in a randomized and blinded fashion. Hence, the investigators performing the stroke surgery, collecting the brain tissue, and quantifying cerebral infarction were unaware of the treatment groups. The treatment groups were randomized using the = RAND-BETWEEN(1,4) function in Microsoft Excel to avoid randomization bias. For mice that were allowed to recover from anesthesia in a heated chamber during the 120-min ischemic period, the mortality rate after dMCAO was 1 out of 144 mice; for mice that remained anesthetized during the 120-min ischemic period to ensure that the temperature maintained in the normothermic range, the mortality rate was 16 out of 52 mice (mortality under this protocol was evenly distributed among the treatment groups). In addition, the mortality rate was 1 out of 5 mice for hyperthermia and 0 out of 10 mice for hypothermia. Other than the mice that died (as indicated by the mortality rate), no animal was excluded from the data analysis.

### Statistical analysis

Data are presented as the mean ± SEM. The infarct areas of the coronal sections across the brain were compared with either 1-way or 2-way repeated measures ANOVA (matching brain sections from the same animal), followed by Holm-Sidak’s multiple comparisons test. The infarct volumes were compared using 1-way ANOVA followed by Holm-Sidak’s multiple comparisons test. The density and spread of FJ+ neurons were compared using 1-way ANOVA or Student’s t test. The changes to body temperature over time were analyzed by 2-way repeated measures ANOVA (matching different time points from the same animal), followed by Holm-Sidak’s multiple comparisons test. Animal behavior was compared by 2-way repeated measures ANOVA (matching behavioral measurements from the same animal) followed by Fisher’s LSD test.

## Results

### Time course of the cerebral infarction after dMCAO

**Fig 1:**
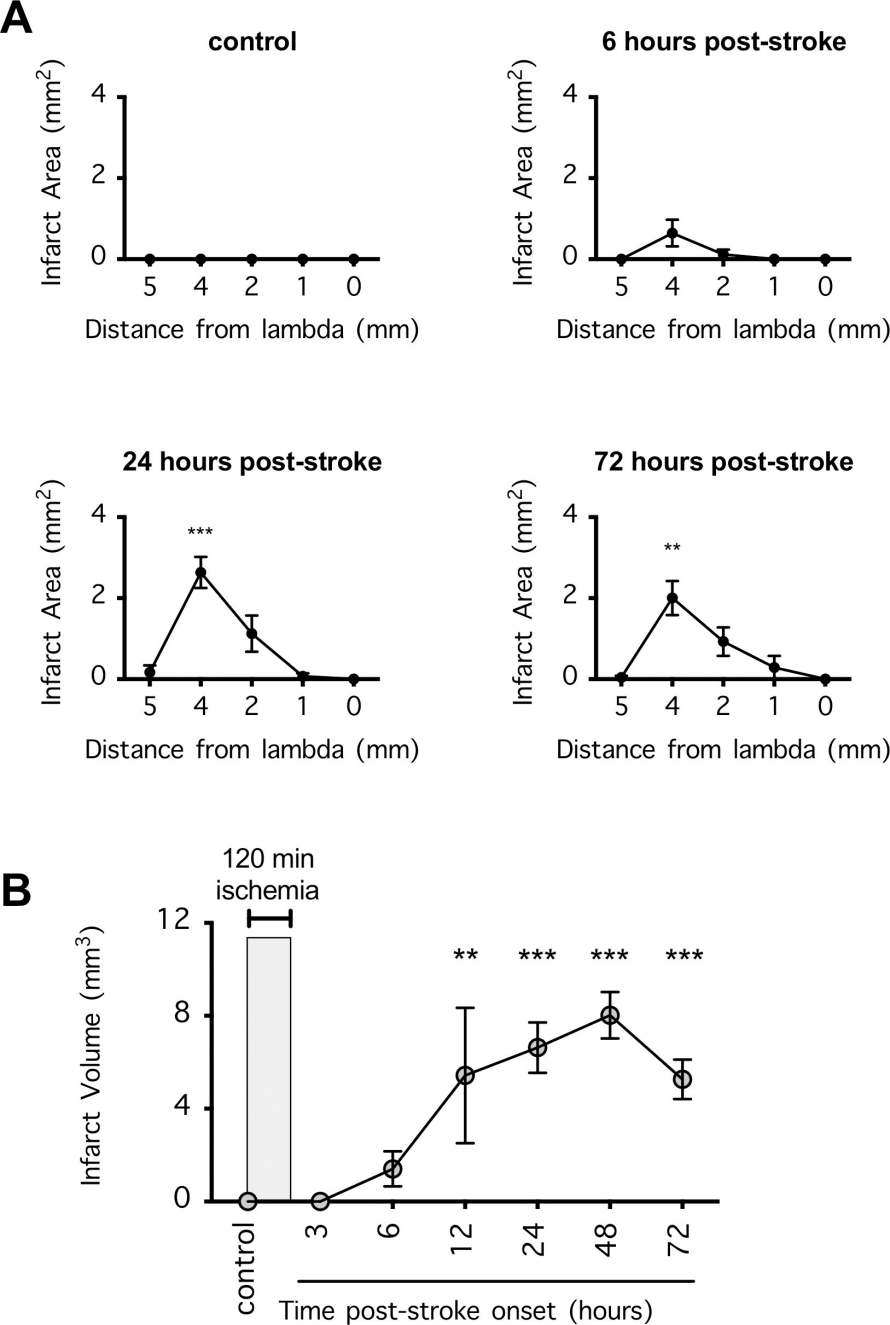
Cerebral infarction after distal middle cerebral arterial occlusion (dMCAO) in mice. (**A**) Mice were subjected to 120 min dMCAO, and 6, 24, and 72 h after ischemia onset, their brains were collected and coronally sectioned to determine the infarct area via 2,3,5-triphenyltetrazolium chloride staining. n = 15 per time point. The infarct areas were compared by 1-way repeated measures ANOVA (matching coronal sections from the same mouse) followed by Holm-Sidak’s multiple comparisons test. **P<0.01 and ***P<0.001 indicate significant differences compared to the noninfarcted sections. (**B**) The total infarct volume extrapolated from **A** (n = 15 per time point) including data from additional time points (3, 12, and 48 h post-ischemia; n = 5 for these time points). The infarct volumes were compared by 1-way ANOVA followed by Holm-Sidak’s multiple comparisons test. **P<0.01 and ***P<0.001 indicate significant differences compared to the control. In A and B, the error bars indicate the mean ± SEM.

**Fig 2:**
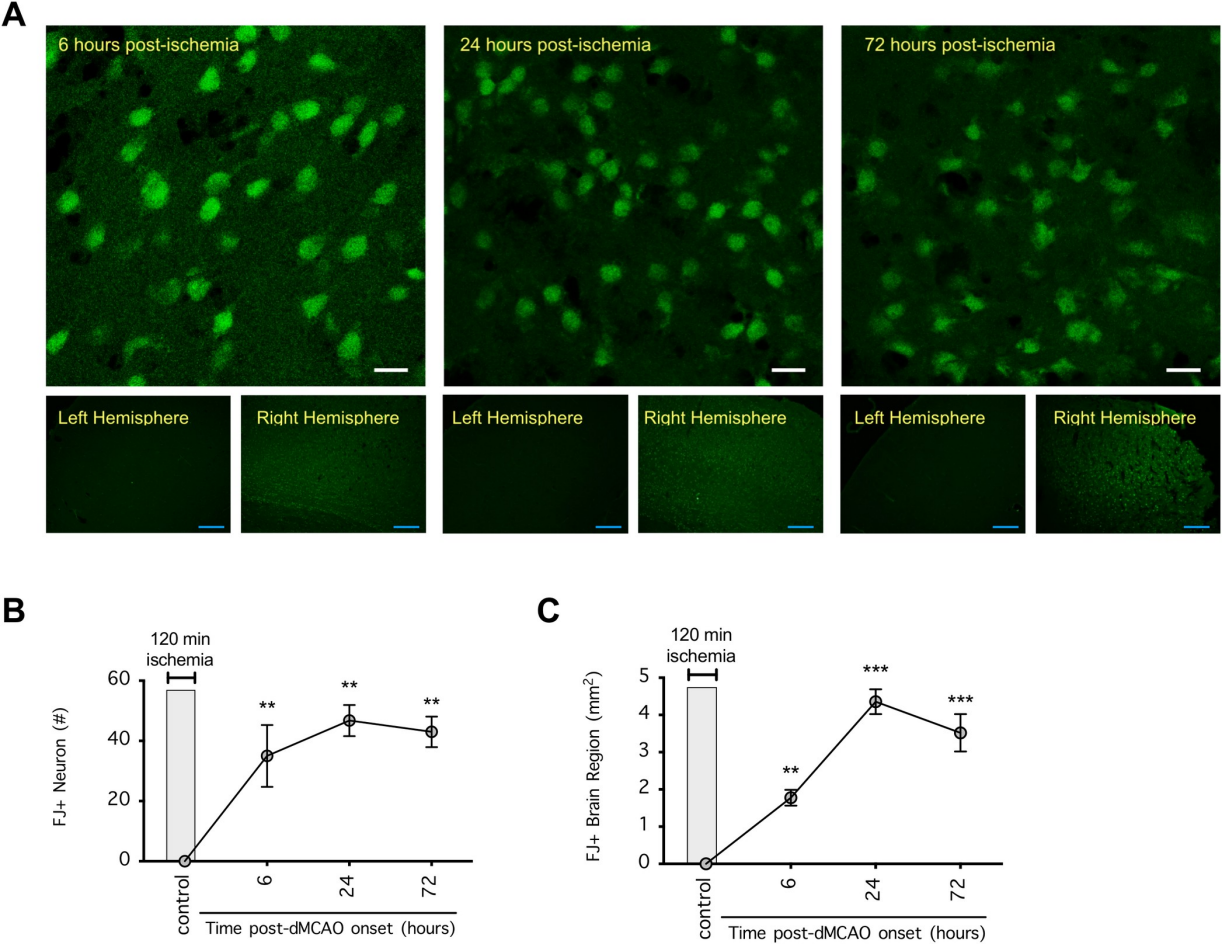
Neurodegeneration after distal middle cerebral arterial occlusion (dMCAO) in mice. (**A**) Representative images of Fluoro-Jade (FJ)-stained brain slices. Mice were subjected to 120 min dMCAO, and 6, 24, and 72 h after ischemia onset, their brains were coronally sectioned and stained with FJ B to determine the density [*top panel (scale bar = 10* μ*m)*, # of FJ+ neurons per 106.7μm x 106.7μm imaged area of the ischemic core] and spread [*bottom panel (scale bar = 200* μ*m)*, FJ+ brain region] of neurodegeneration. (**B**) Summarized data from **A** showing neurodegeneration in the ischemic core over time. (**C**) Summarized data from **A** showing the spread of neurodegeneration over time. In **B** and **C**, error bars indicate the mean ± SEM, and n = 4 per time point. Data were compared by 1-way ANOVA followed by Holm-Sidak’s multiple comparisons test. **P<0.01 and ***P<0.001 indicate significant differences compared to the noninfarcted sections.

To follow the time course of the focal ischemic injury after 120 min of dMCAO, we euthanized the mice at 3, 6, 12, 24, 48, and 72 h post-ischemia onset to quantify cerebral infarction. This stroke model produced prominent infarction at the rostral cortical brain regions +4 mm from the lambda, and when the infarct fully matured, it extended to more caudal cortical regions at +1 mm from the lambda (**Fig 1A**). Importantly, no cerebral infarction was found 3 h post-ischemia, suggesting that most of the damage occurred after the 120-min ischemic period (**Fig 1B**). Cerebral infarction became evident by 6 h post-ischemia (albeit smaller in size) and plateaued at 24–48 h post-ischemia (P<0.001) (**Fig 1B**). Likewise, degenerating neurons were evident by 6 h post-ischemia (**Fig 2A**), and their density in the ischemic core and total area of occupancy plateaued at 24–48 h post-ischemia (**Fig 2B and 2C**). These data suggest that neuroprotection could be possible if stroke damage was intervened within 6 h post-ischemia.

### Effect of hypothermia on the ischemic damage induced by dMCAO

**Fig 3:**
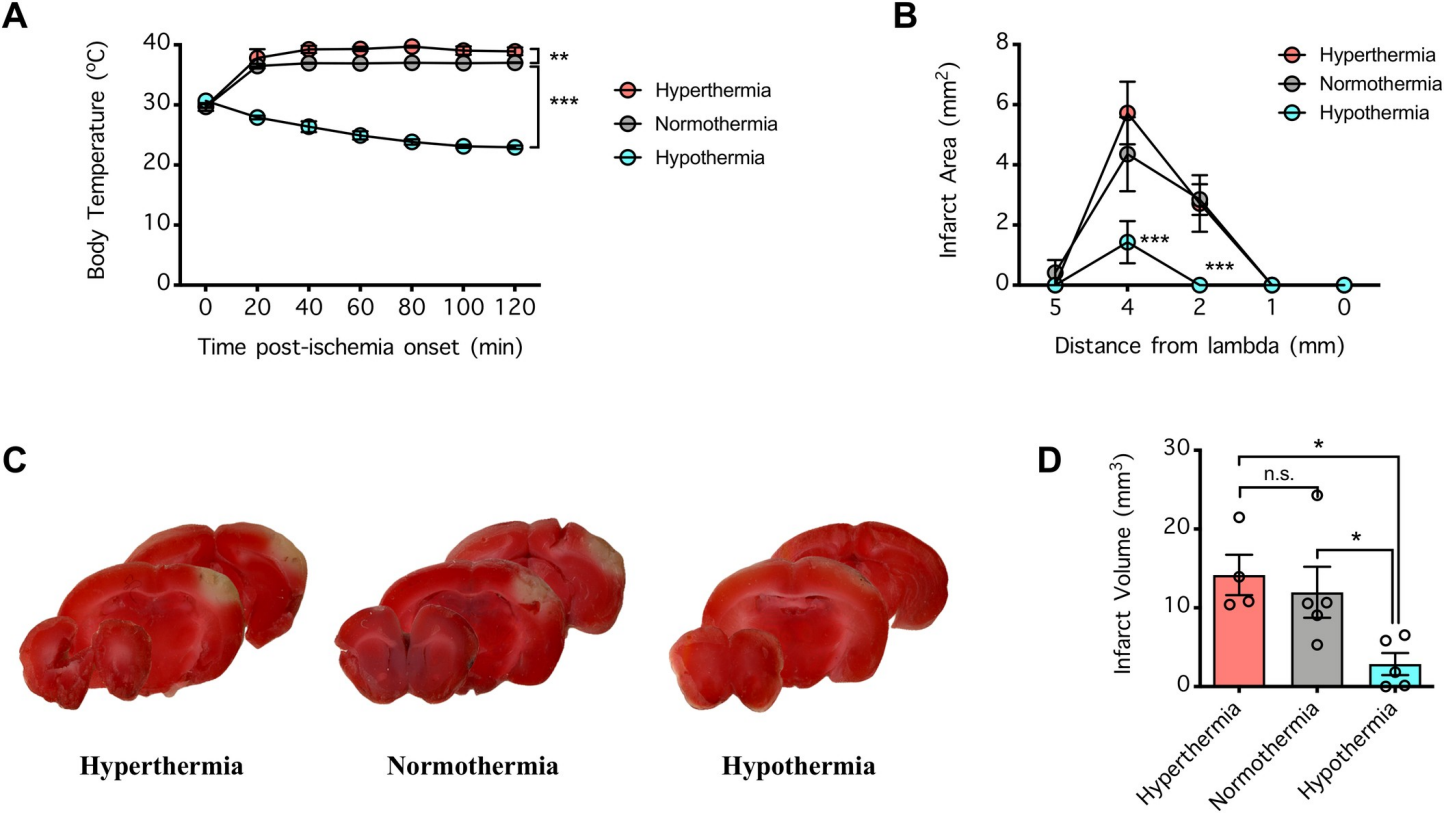
Effect of hypothermia on brain infarcts in the mice subjected to distal middle cerebral arterial occlusion (dMCAO). (**A**) Mice were subjected to 120 min dMCAO, and during the ischemic period, their body temperatures were maintained in the hyperthermic, normothermic, or hypothermic ranges under isoflurane anesthesia via a heating pad. Body temperature was recorded via a rectal probe during the 120-min ischemic period. Data were compared by 2-way repeated measures ANOVA (matching body temperature from the same animal) followed by Holm-Sidak’s multiple comparisons test. **P<0.01 and ***P<0.001 indicate significant differences compared to normothermia. (**B**) At 24 h post-ischemia, the brains of the mice from **A** were collected and coronally sectioned to determine the infarct area via 2,3,5-triphenyltetrazolium chloride (TTC) staining. The infarct areas were compared by 2-way repeated measures ANOVA (matching coronal sections from the same mouse) followed by Holm-Sidak’s multiple comparisons test. ***P<0.001 indicates a significant difference compared to normothermia. (**C**) Representative TTC-stained brain slices from **B**. (**D**) Total infarct volume extrapolated from **B**. The infarct volumes were compared by 1-way ANOVA followed by Holm-Sidak’s multiple comparisons test. *P<0.05 indicates a significant difference; n.s. indicates no significant difference. In **A**, **B**, and **D**, n = 5 per group, and error bars indicate the mean ± SEM.

**Fig 4:**
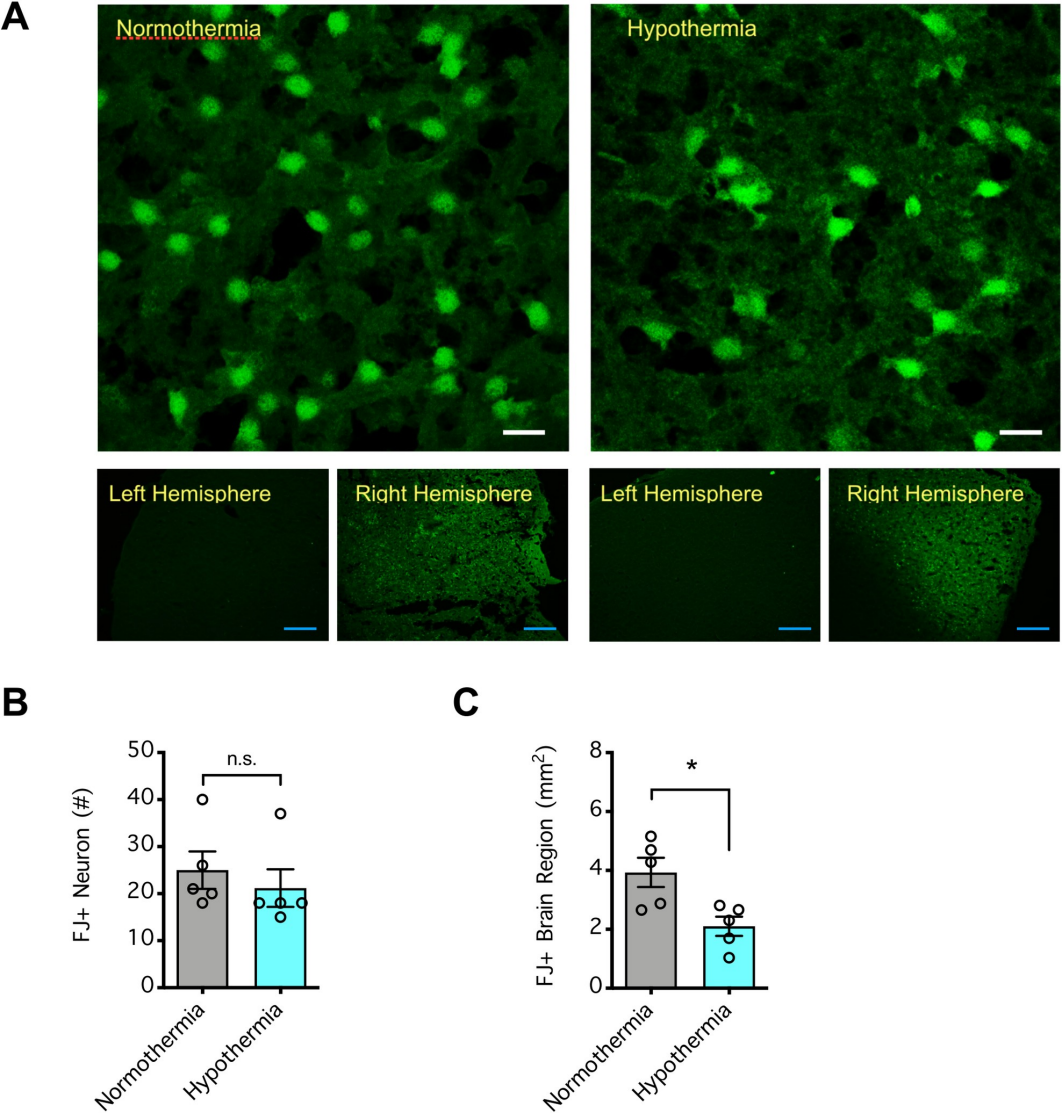
Neuroprotective effect of hypothermia in the mice subjected to distal middle cerebral arterial occlusion (dMCAO). (**A**) Representative images of Fluoro-Jade (FJ)-stained brain slices. Mice were subjected to 120 min dMCAO, and during the ischemic period, their body temperatures were either normothermic or hypothermic. At 72 h after ischemia onset, their brains were coronally sectioned and stained with FJ B to determine the density [*top panel (scale bar = 10 μm)*, # of FJ+ neurons per 106.7μm x 106.7μm imaged area of the ischemic core] and spread [*bottom panel (scale bar = 200* μ*m)*, FJ+ brain region] of neurodegeneration. (**B**) Summarized data from **A** showing neurodegeneration in the ischemic core over time. (**C**) Summarized data from **A** showing the spread of neurodegeneration over time. In **B** and **C**, error bars indicate the mean ± SEM, and n = 5 per group. Data were compared by Student’s t test. *P<0.05 indicates a significant difference; n.s. indicates no significant difference.

To confirm that brain infarction could be salvaged after intervention by neuroprotective strategies, we manipulated the body temperature of the mice during the 120-min ischemic period of dMCAO via a heating pad to maintain normothermia or to achieve hyperthermia or hypothermia. The body temperature of each mouse typically dropped to ~30°C by the end of the surgical preparation (immediately prior to/after vascular occlusion), after which placement onto the heating pad quickly (within 20 min) readjusted the body temperature to normothermic (~37°C) or hyperthermic (39–40°C) levels depending on the setting of the heating pad (**Fig 3A**). However, body temperature dropped to the hypothermic range when isoflurane anesthesia (1%) was maintained without the heating pad (P<0.001, compared to normothermia) (**Fig 3A**). Under these experimental conditions, hypothermia was strongly protective, as it dramatically decreased the infarct area across brain sections (P<0.001, compared to normothermia) and the total infarct volume (P<0.05, compared to normothermia) (**Fig 3B–3D**). Although this strategy could not appreciably decrease the density of the degenerating neurons in the ischemic core (**Fig 4A and 4B**), it decreased the area of neurodegeneration in the ischemic hemisphere of the brain (**Fig 4C**). The protective effect of hypothermia is consistent with the hypothesis that stroke damage caused by dMCAO can be prevented by neuroprotective measures.

### Effects of NMDAR antagonism on mouse behavior and body temperature

**Fig 5:**
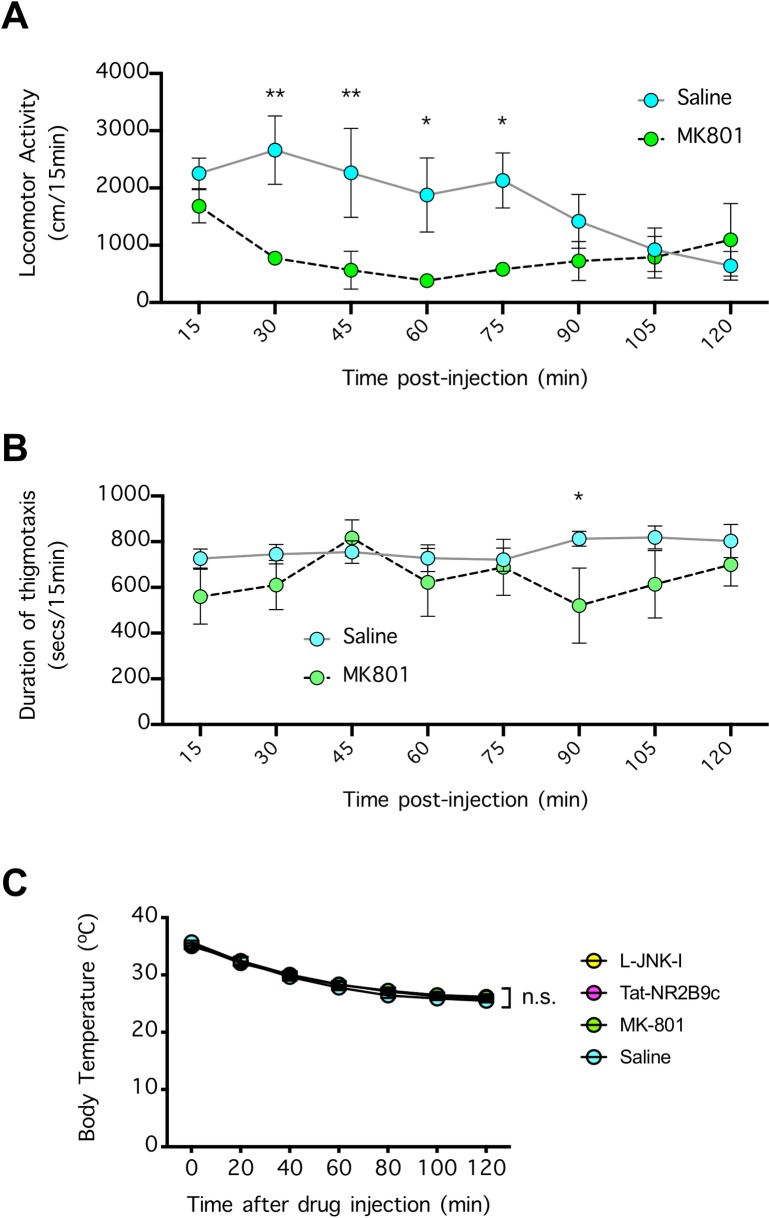
Effect of NMDAR antagonist on the open field behavior and body temperature of the mice. (**A**) Mice were injected (i.p.) with saline (4 ml/kg) or MK801 (4 mg/kg), and the locomotor activity was investigated in an open field test chamber. n = 4 per group. Data were compared by 2-way repeated measures ANOVA (matching locomotion over time from the same animal) followed by Fisher’s LSD test. *P<0.05 and **P<0.01 indicate significant differences compared to the saline-treated mice. (**B**) Thigmotaxis of mice in **A** was determined by measuring the time they spent near the chamber wall. n = 4 per group. Data were compared by 2-way repeated measures ANOVA (matching thigmotaxis measured from the same animal) followed by Fisher’s LSD test. *P<0.05 indicates a significant difference compared to the saline-treated mice. (**C**) Mice were injected (i.p.) with MK801 (4 mg/kg), Tat-NR2B9c (8 mg/kg), L-JNKI-1 (8 mg/kg), or vehicle (saline at 4 ml/kg), and after light sedation by 1% isoflurane, the body temperature was measured by a rectal probe. n = 5 per group. Data were compared by 2-way repeated measures ANOVA (matching body temperature from the same animal); n.s. indicates no significant difference. In **A**-**C**, error bars indicate the mean ± SEM.

To determine whether the prototypical NMDAR antagonist MK801 can be efficiently delivered and exert pharmacological effects in the mouse brain using the dose and route of administration in our experimental setting, we measured mouse behavior in an open field test chamber and found that MK801 (4 mg/kg, i.p.) rapidly decreased the locomotor activity in these mice soon after injection (P<0.001 compared to the saline-treated mice) and had a marginal inhibitory effect on the duration of thigmotaxis (however, the difference was only significant at one time point: 90 min post-injection, when P<0.05 compared to the saline-treated mice) (**Fig 5A and 5B**). Given that MK801 can decrease body temperature in gerbils to achieve neuroprotection [22–24], we examined whether NMDAR antagonism affected the body temperature of the mice. In contrast to the results in gerbils, none of the treatments tested had an effect on body temperature in the mice (P>0.05) (**Fig 5C**).

### Lack of neuroprotection by NMDAR antagonism against dMCAO

**Fig 6:**
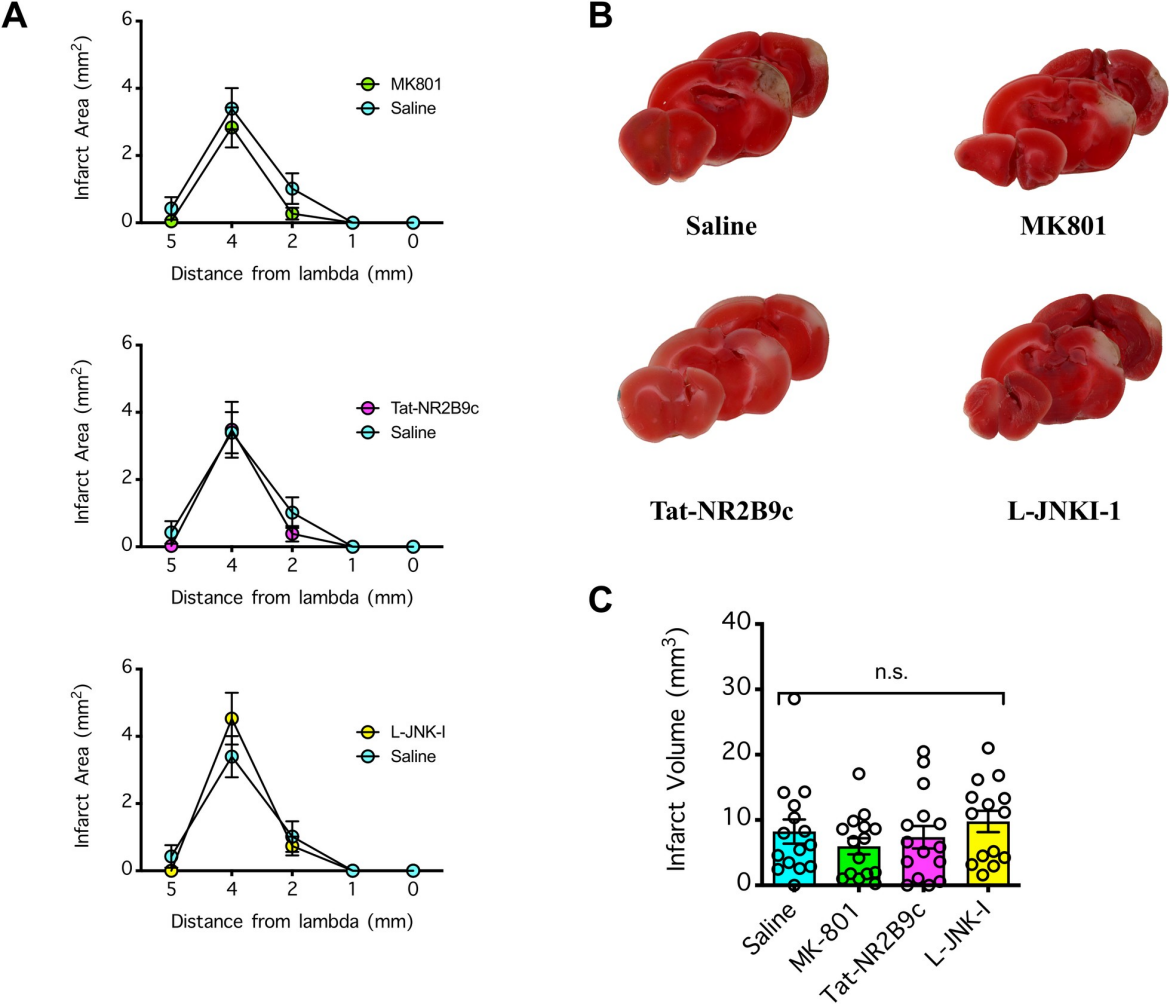
Effect of NMDAR antagonism on brain infarction induced by distal middle cerebral arterial occlusion (dMCAO) in awake mice. (**A**) Mice were injected (i.p.) with MK801 (4 mg/kg), Tat-NR2B9c (8 mg/kg), L-JNKI-1 (8 mg/kg), or vehicle (saline at 4 ml/kg) 30 min prior to induction of focal ischemia by 120 min dMCAO. During the 120 min ischemic period, the mice were allowed to recover from anesthesia in a heated chamber, and 22 h post-reperfusion, their brains were collected and coronally sectioned to determine the infarct areas via 2,3,5-triphenyltetrazolium chloride (TTC) staining. Comparison by 2-way repeated measures ANOVA (matching coronal sections from the same mouse) found no significant difference among the treatment groups. (**B**) Representative TTC-stained brain slices from **A**. (**C**) Total infarct volume extrapolated from **A**. The infarct volumes were compared by 1-way ANOVA; n.s. indicates no significant difference. In **A** and **C**, n = 14–15 per treatment group, and error bars indicate the mean ± SEM.

**Fig 7:**
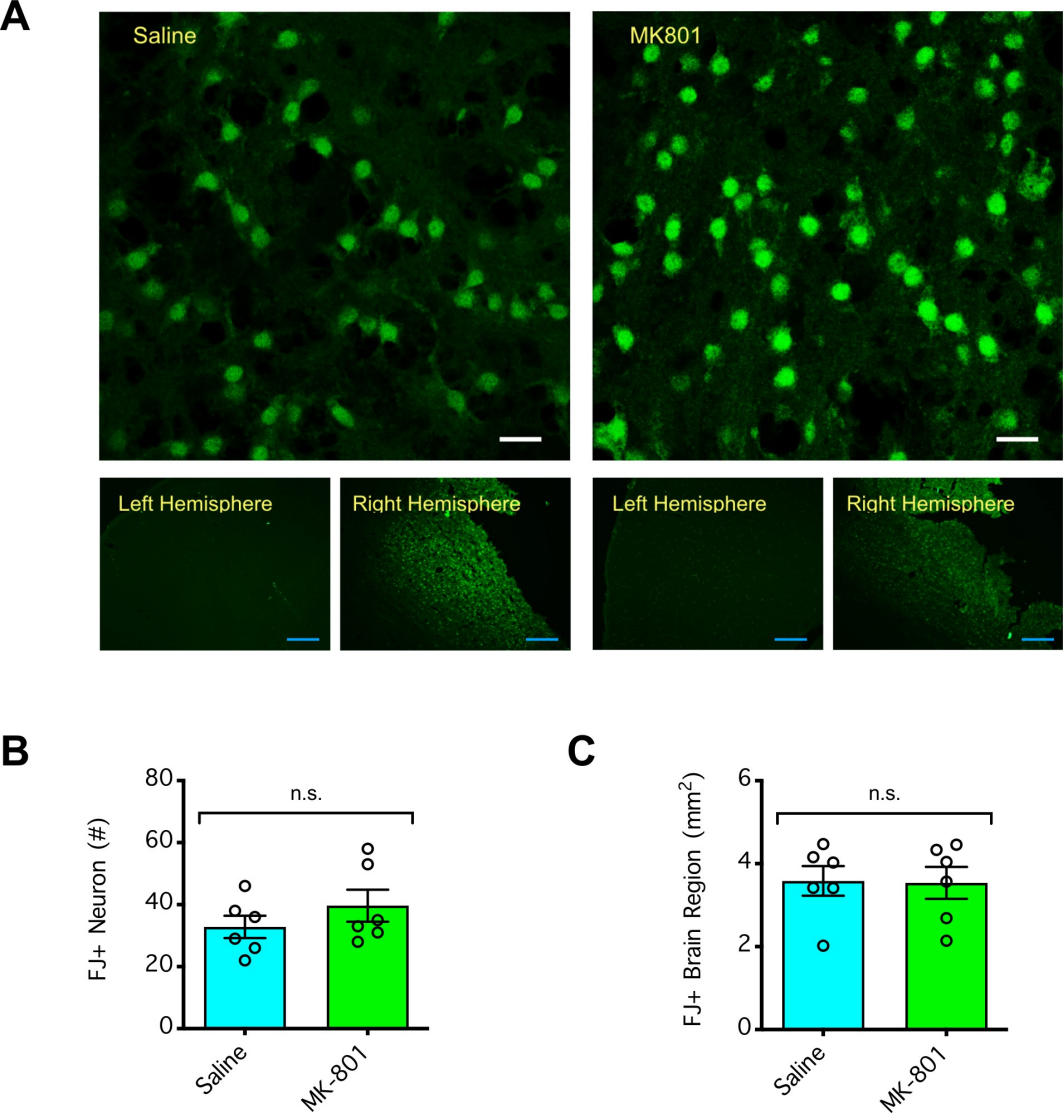
Effect of NMDAR antagonism on neurodegeneration induced by distal middle cerebral arterial occlusion (dMCAO) in awake mice. (**A**) Representative images of Fluoro-Jade (FJ)-stained brain slices. Mice were injected (i.p.) with MK801 (4 mg/kg), Tat-NR2B9c (8 mg/kg), L-JNKI-1 (8 mg/kg), or vehicle (saline at 4 ml/kg) 30 min prior to induction of focal ischemia by 120 min dMCAO. During the 120 min ischemic period, the mice were allowed to recover from anesthesia in a heated chamber, and 72 h post-reperfusion, their brains were coronally sectioned and stained with FJ B to determine the density [*top panel (scale bar = 10* μ*m)*, # of FJ+ neurons per 106.7μm x 106.7μm imaged area of the ischemic core] and spread [*bottom panel (scale bar = 200* μ*m)*, FJ+ brain region] of neurodegeneration. (**B**) Summarized data from **A** showing neurodegeneration in the ischemic core over time. (**C**) Summarized data from **A** showing the spread of neurodegeneration over time. In **B** and **C**, the error bars indicate the mean ± SEM, and n = 6 per group. Data were compared by Student’s t test. n.s. indicates no significant difference.

**Fig 8:**
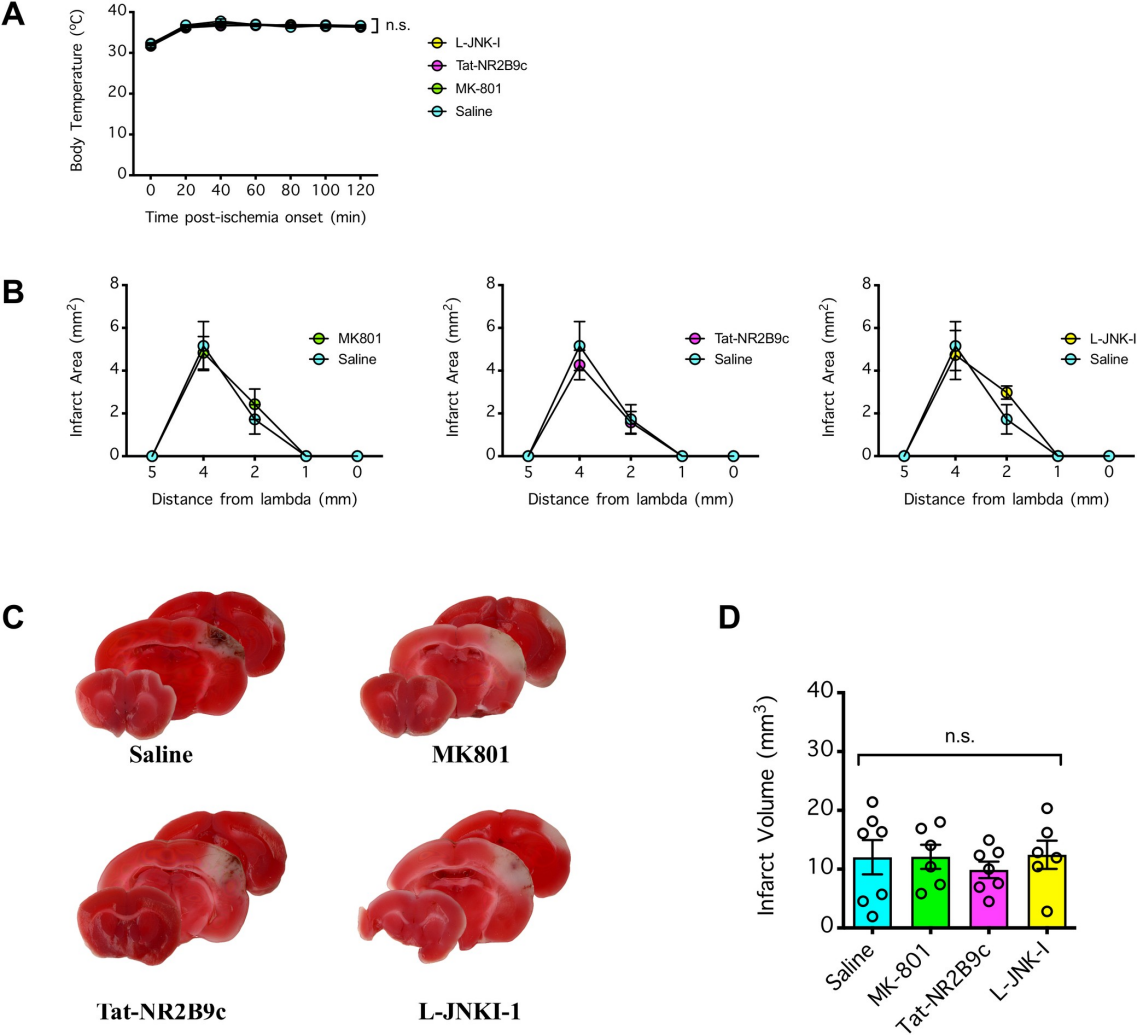
Effect of NMDAR antagonism against distal middle cerebral arterial occlusion (dMCAO) in the anesthetized mice under temperature-controlled normothermia. (**A**) Mice were injected (i.p.) with MK801 (4 mg/kg), Tat-NR2B9c (8 mg/kg), L-JNKI-1 (8 mg/kg), or vehicle (saline at 4 ml/kg) 30 min prior to induction of focal ischemia by 120 min dMCAO. During the 120 min ischemic period, the mice remained under surgical anesthesia, and the body temperature was tightly controlled via a rectal probe coupled automatically adjusting heating pad. The body temperature over the ischemic period was recorded. Data were compared by 2-way repeated measures ANOVA (matching body temperature from the same animal); n.s. indicates no significant difference. (**B**) Brains of the mice from A were collected 22 h after reperfusion and coronally sectioned to determine the infarct area via 2,3,5-triphenyltetrazolium chloride (TTC) staining. Comparison by 2-way repeated measures ANOVA (matching coronal sections from same mouse) found no significant difference among the treatment groups. (**C**) Representative TTC-stained brain slices from **B**. (**D**) The total infarct volume extrapolated from **B**. The infarct volumes were compared by 1-way ANOVA; n.s. indicates no significant difference. In **A**, **B** and **D**, n = 6–7 per treatment group, and error bars indicate the mean ± SEM.

Given that MK801 at 4 mg/kg was more than sufficient to achieve neuroprotection against stroke induced by MCAO (with a 50% effective dose [ED50] of 0.3 mg/kg and substantial neuroprotection by 3 mg/kg [4]), we expected it would be neuroprotective against stroke induced by dMCAO. To test this hypothesis, we injected (i.p.) mice with MK801 (4 mg/kg), Tat-NR2B9c (8 mg/kg), L-JNKI-1 (8 mg/kg), or vehicle (saline at 4 ml/kg) 30 min prior to induction of transient 120-min dMCAO. During the ischemic period, the mice were either allowed to recover from anesthesia (**Figs 6 and 7**) or remained anesthetized to maintain their body temperature in the normothermic range (**Fig 8**). Consistent with our time course data (**Fig 1A and 1B**), the mice that were allowed to recover during the 120 min ischemic period developed a small infarct (on average ~7–8 mm^3^ in volume, with a maximum infarct area of ~3 mm^2^ at +4 mm from the lambda) (**Fig 6A–6C**). Nevertheless, none of the therapeutic treatments had neuroprotective effects compared to the control treatment with saline (P>0.05) (**Figs 6A–6C and 7A–7C**). Likewise, consistent with our temperature control experiments (**Fig 3**), the mice that remained anesthetized with a body temperature maintained in the normothermic range during the 120-min ischemic period developed a large cortical infarct (on average ~12 mm^3^ in volume, with maximum infarct area of ~4–5 mm^2^ at +4 mm from the lambda) (**Fig 8B–8D**). Again, none of the therapeutic treatments had an effect on the body temperature of the mice during the ischemic period (P>0.05) (**Fig 8A**) nor did they have a neuroprotective effect compared to the control treatment with saline (P>0.05) (**Fig 8B–8D**). Our data showed the reproducibility of the transient 120-min dMCAO, either with the mice awake or under anesthesia with a normothermic temperature during ischemia, in producing cortical infarction that averaged ~7–8 mm^3^ and ~12 mm^3^, respectively. Unexpectedly, none of the anti-excitotoxic agents tested, all of which were administered at doses that were neuroprotective in the MCAO stroke model, were neuroprotective against stroke induced by dMCAO.

## Discussion

Animal models are often used for preclinical assessment of drugs developed to treat stroke, which still lacks effective therapeutic options available for clinical use. The most popular focal ischemic stroke models are the MCAO models, which produce a large hemispheric infarct covering cortical and many subcortical brain structures. In these models, the extent of the damage can vary depending on the depth of the suture insertion, the thickness of the occluder used, and the duration of ischemia [28]. Notably, damage to the striatum can limit animal mobility, and damage to the hypothalamus can cause hyperthermia to further exacerbate ischemic damage. Therefore, the beneficial effect of drugs in these models can be partly due to other pharmacological effects independent of neuroprotection *per se*. While these pathophysiological features associated with large infarcts in the MCAO models in some ways resemble those of stroke in the clinic, a stroke model with minimal pathophysiological effects aside from neuronal damage would be useful for determining the neuroprotective efficacy of drugs. In the present study, we showed that the ischemic stroke induced by dMCAO produced a unilateral cerebral infarction that is confined to the dorsal cortex, sparing the striatum, hypothalamus, and other subcortical structures. Similar to that of the MCAO models, the cortical damage induced by transient dMCAO is delayed, occurring hours after ischemia has already ended, and progressive, increasing in size over time. These features suggest that the infarction caused by dMCAO is an active process (as opposed to what would be expected from a passive traumatic insult) that would be salvageable by neuroprotective measures. Consistent with this hypothesis, hypothermia was strongly neuroprotective against cerebral infarction in this model. Moreover, we found that the state of the animal during the ischemic period can affect the infarct volumes. Specifically, mice that were kept anesthetized while maintaining body temperature at the normothermic range during the ischemic period had a large infarct (on average ~12 mm^3^ in volume, with maximum infarct area of ~4–5 mm^2^ at +4 lambda) associated with a high mortality rate (16 out of 52 mice died after dMCAO). This finding is in stark contrast to the results in the mice that were allowed to recover from anesthesia during the ischemic period; these mice had a small infarct on average of ~7–8 mm^3^ in volume, with a maximum infarct area of ~3 mm^2^ at +4 lambda and almost no mortality (1 out of 144 mice died after dMCAO). Therefore, the dMCAO model can be fine-tuned to produce the desired stroke outcome for preclinical drug assessment.

Despite the overwhelming experimental evidence that excitotoxicity is the primary mechanism of ischemic neuronal injury, none of the neuroprotective drugs that inhibit excitotoxicity could improve stroke outcomes in large clinical trials. The reason for clinical failure is often multifactorial, but the collective failure of so many trials raises the question of whether the animal model used is suitable. Given that the majority of the focal ischemia experiments that evaluated the neuroprotective efficacy of NMDAR antagonism used one of the MCAO models, we asked whether the prototypical NMDAR antagonist MK801 would be neuroprotective against stroke damage caused by dMCAO. To test this hypothesis, we injected mice with MK801 at a dose that was more than sufficient to achieve neuroprotection against stroke induced by MCAO. We found that MK801 readily depressed locomotor activity in the mice, suggesting efficient central delivery and pharmacological action in the brain; nevertheless, the drug failed to protect the mice against the cerebral infarction induced by dMCAO, either in awake mice (with a small infarct and no mortality) or in anesthetized mice under normothermic temperature control (with a large infarct and some mortality). These data thus suggest that NMDAR antagonists, at a dose and treatment regimen that was effective in the animal MCAO models, would not be effective against this type of ischemic insult in the clinic.

The clinical failure of conventional NMDAR inhibitors has prompted the development of therapeutic peptides and peptide mimetics that target death-signaling pathways downstream of the NMDAR [37, 38]. The first of these to reach clinical trials is Tat-NR2B9c [39], which interferes with the coupling of NMDAR and nNOS, thereby preventing the production of neurotoxic nitric oxide during stroke [34]. As Tat-NR2B9c targets only the NMDAR-nNOS signaling pathway, it is expected to have far fewer side effects than conventional inhibitors that block NMDAR at its cell surface level [34]. Importantly, this peptide has shown early success in a clinical trial, in which it reduced the number of iatrogenic ischemic infarcts in patients undergoing endovascular repair for intracranial aneurysm [39]. Another therapeutic peptide in development is the JNK-inhibiting peptide (JNKI-1; also known as Tat-JBD20) [40, 41]. Notably, although JNK is activated soon after NMDAR stimulation and/or stroke, it remains activated for up to 24 h after stroke. Therefore, compared to conventional NMDAR inhibitors and Tat-NR2B9c, JNKI-1 is expected to show neuroprotection when administration is delayed for many hours after stroke. Nevertheless, given that these peptides target the death-signaling cascades downstream of the NMDAR, they would not be expected to be effective in models where pretreatment of NMDAR antagonists is not effective. Indeed, these peptides also failed to protect against cerebral infarction by dMCAO in this study.

Consistent with the results from this study, NMDAR antagonism was shown to lack neuroprotection in rats [17–19] and piglets [20] subjected to severe global ischemia. In gerbils, NMDAR antagonism by MK801 was neuroprotective only when the drug produced marked hypothermia but not when the body temperature was maintained in the normothermic range [22–24]. The lack of neuroprotection by NMDAR antagonism in the dMCAO and other ischemia models is reminiscent of the clinical failures seen with these neuroprotective drugs but does not overcome the extensive evidence that NMDAR antagonism is strongly neuroprotective in many animal models of ischemia, including the MCAO models. Thus, further research is needed before the most suitable drug dose, treatment regimen, and stroke type for neuroprotection by NMDAR antagonism can be determined for stroke patients in the clinic.

## Supporting information

S1 Fig Photographic demonstration of the distal middle cerebral artery (dMCA) and its occlusion. **(A)** Representative photo of a mouse dMCA imaged following craniectomy. **(B)** Photo image showing a needle placed under the arterial branch to be occluded by suture ligation.
(PDF)

S1 Data (XLSX)

## References

[1] Lai TW, Zhang S, Wang YT. Excitotoxicity and stroke: Identifying novel targets for neuroprotection. Progress in neurobiology. 2014;115:157–88. 10.1016/j.pneurobio.2013.11.006 WOS:000334084100009. 24361499

[2] Park CK, Nehls DG, Graham DI, Teasdale GM, McCulloch J. The glutamate antagonist MK-801 reduces focal ischemic brain damage in the rat. Annals of neurology. 1988;24(4):543–51. Epub 1988/10/01. 10.1002/ana.410240411 . 2853604

[3] Gill R, Brazell C, Woodruff GN, Kemp JA. The neuroprotective action of dizocilpine (MK-801) in the rat middle cerebral artery occlusion model of focal ischaemia. Br J Pharmacol. 1991;103(4):2030–6. Epub 1991/08/01. 10.1111/j.1476-5381.1991.tb12371.x PubMed Central PMCID: PMC1908204. 1912992 PMC1908204

[4] Hatfield RH, Gill R, Brazell C. The dose-response relationship and therapeutic window for dizocilpine (MK-801) in a rat focal ischaemia model. European journal of pharmacology. 1992;216(1):1–7. Epub 1992/05/27. 10.1016/0014-2999(92)90201-e . 1526248

[5] Buchan AM, Slivka A, Xue D. The effect of the NMDA receptor antagonist MK-801 on cerebral blood flow and infarct volume in experimental focal stroke. Brain research. 1992;574(1–2):171–7. Epub 1992/03/06. 10.1016/0006-8993(92)90814-p . 1386274

[6] Lo EH, Matsumoto K, Pierce AR, Garrido L, Luttinger D. Pharmacologic reversal of acute changes in diffusion-weighted magnetic resonance imaging in focal cerebral ischemia. Journal of cerebral blood flow and metabolism: official journal of the International Society of Cerebral Blood Flow and Metabolism. 1994;14(4):597–603. Epub 1994/07/01. 10.1038/jcbfm.1994.74 . 8014206

[7] Margaill I, Parmentier S, Callebert J, Allix M, Boulu RG, Plotkine M. Short therapeutic window for MK-801 in transient focal cerebral ischemia in normotensive rats. Journal of cerebral blood flow and metabolism: official journal of the International Society of Cerebral Blood Flow and Metabolism. 1996;16(1):107–13. Epub 1996/01/01. 10.1097/00004647-199601000-00013 . 8530543

[8] Ma J, Endres M, Moskowitz MA. Synergistic effects of caspase inhibitors and MK-801 in brain injury after transient focal cerebral ischaemia in mice. Br J Pharmacol. 1998;124(4):756–62. Epub 1998/08/05. 10.1038/sj.bjp.0701871 9690868 PMC1565432

[9] Ozyurt E, Graham DI, Woodruff GN, McCulloch J. Protective effect of the glutamate antagonist, MK-801 in focal cerebral ischemia in the cat. Journal of cerebral blood flow and metabolism: official journal of the International Society of Cerebral Blood Flow and Metabolism. 1988;8(1):138–43. Epub 1988/02/01. 10.1038/jcbfm.1988.18 . 2892846

[10] Park CK, Nehls DG, Graham DI, Teasdale GM, McCulloch J. Focal cerebral ischaemia in the cat: treatment with the glutamate antagonist MK-801 after induction of ischaemia. Journal of cerebral blood flow and metabolism: official journal of the International Society of Cerebral Blood Flow and Metabolism. 1988;8(5):757–62. Epub 1988/10/01. 10.1038/jcbfm.1988.124 . 2901425

[11] Rod MR, Auer RN. Pre- and post-ischemic administration of dizocilpine (MK-801) reduces cerebral necrosis in the rat. Can J Neurol Sci. 1989;16(3):340–4. Epub 1989/08/01. 10.1017/s031716710002919x . 2670155

[12] Rod MR, Whishaw IQ, Auer RN. The relationship of structural ischemic brain damage to neurobehavioural deficit: the effect of postischemic MK-801. Can J Psychol. 1990;44(2):196–209. Epub 1990/06/01. 10.1037/h0084242 . 2200595

[13] Swan JH, Meldrum BS. Protection by NMDA antagonists against selective cell loss following transient ischaemia. Journal of cerebral blood flow and metabolism: official journal of the International Society of Cerebral Blood Flow and Metabolism. 1990;10(3):343–51. Epub 1990/05/01. 10.1038/jcbfm.1990.63 . 2158499

[14] McDonald JW, Silverstein FS, Johnston MV. MK-801 protects the neonatal brain from hypoxic-ischemic damage. European journal of pharmacology. 1987;140(3):359–61. Epub 1987/08/21. 10.1016/0014-2999(87)90295-0 . 2820765

[15] Ikonomidou C, Mosinger JL, Olney JW. Hypothermia enhances protective effect of MK-801 against hypoxic/ischemic brain damage in infant rats. Brain research. 1989;487(1):184–7. Epub 1989/05/15. 10.1016/0006-8993(89)90956-6 . 2546648

[16] Olney JW, Ikonomidou C, Mosinger JL, Frierdich G. MK-801 prevents hypobaric-ischemic neuronal degeneration in infant rat brain. The Journal of neuroscience: the official journal of the Society for Neuroscience. 1989;9(5):1701–4. Epub 1989/05/01. 10.1523/JNEUROSCI.09-05-01701.1989 . 2656934 PMC6569821

[17] Buchan A, Li H, Pulsinelli WA. The N-methyl-D-aspartate antagonist, MK-801, fails to protect against neuronal damage caused by transient, severe forebrain ischemia in adult rats. The Journal of neuroscience: the official journal of the Society for Neuroscience. 1991;11(4):1049–56. Epub 1991/04/01. 10.1523/JNEUROSCI.11-04-01049.1991 . 2010804 PMC6575368

[18] Nellgard B, Gustafson I, Wieloch T. Lack of protection by the N-methyl-D-aspartate receptor blocker dizocilpine (MK-801) after transient severe cerebral ischemia in the rat. Anesthesiology. 1991;75(2):279–87. Epub 1991/08/01. 10.1097/00000542-199108000-00016 . 1859015

[19] Nellgard B, Wieloch T. Postischemic blockade of AMPA but not NMDA receptors mitigates neuronal damage in the rat brain following transient severe cerebral ischemia. Journal of cerebral blood flow and metabolism: official journal of the International Society of Cerebral Blood Flow and Metabolism. 1992;12(1):2–11. Epub 1992/01/01. 10.1038/jcbfm.1992.2 . 1345757

[20] LeBlanc MH, Vig V, Smith B, Parker CC, Evans OB, Smith EE. MK-801 does not protect against hypoxic-ischemic brain injury in piglets. Stroke; a journal of cerebral circulation. 1991;22(10):1270–5. Epub 1991/10/01. 10.1161/01.str.22.10.1270 . 1926238

[21] Gill R, Foster AC, Woodruff GN. Systemic administration of MK-801 protects against ischemia-induced hippocampal neurodegeneration in the gerbil. The Journal of neuroscience: the official journal of the Society for Neuroscience. 1987;7(10):3343–9. Epub 1987/10/01. 10.1523/JNEUROSCI.07-10-03343.1987 . 3312511 PMC6569187

[22] Buchan A, Pulsinelli WA. Hypothermia but not the N-methyl-D-aspartate antagonist, MK-801, attenuates neuronal damage in gerbils subjected to transient global ischemia. The Journal of neuroscience: the official journal of the Society for Neuroscience. 1990;10(1):311–6. Epub 1990/01/01. 10.1523/JNEUROSCI.10-01-00311.1990 . 2405111 PMC6570351

[23] Corbett D, Evans S, Thomas C, Wang D, Jonas RA. MK-801 reduced cerebral ischemic injury by inducing hypothermia. Brain research. 1990;514(2):300–4. Epub 1990/04/30. 10.1016/0006-8993(90)91424-f . 2162711

[24] Warner MA, Neill KH, Nadler JV, Crain BJ. Regionally selective effects of NMDA receptor antagonists against ischemic brain damage in the gerbil. Journal of cerebral blood flow and metabolism: official journal of the International Society of Cerebral Blood Flow and Metabolism. 1991;11(4):600–10. Epub 1991/07/01. 10.1038/jcbfm.1991.110 . 1828809

[25] Memezawa H, Zhao Q, Smith ML, Siesjo BK. Hyperthermia nullifies the ameliorating effect of dizocilpine maleate (MK-801) in focal cerebral ischemia. Brain research. 1995;670(1):48–52. Epub 1995/01/23. 10.1016/0006-8993(94)01251-c . 7719723

[26] Alkan T, Kahveci N, Buyukuysal L, Korfali E, Ozluk K. Neuroprotective effects of MK 801 and hypothermia used alone and in combination in hypoxic-ischemic brain injury in neonatal rats. Arch Physiol Biochem. 2001;109(2):135–44. Epub 2002/01/10. 10.1076/apab.109.2.135.4271 . 11780774

[27] Gerriets T, Stolz E, Walberer M, Kaps M, Bachmann G, Fisher M. Neuroprotective effects of MK-801 in different rat stroke models for permanent middle cerebral artery occlusion: adverse effects of hypothalamic damage and strategies for its avoidance. Stroke; a journal of cerebral circulation. 2003;34(9):2234–9. Epub 2003/08/16. 10.1161/01.STR.0000087171.34637.A9 . 12920258

[28] Li F, Omae T, Fisher M. Spontaneous hyperthermia and its mechanism in the intraluminal suture middle cerebral artery occlusion model of rats. Stroke; a journal of cerebral circulation. 1999;30(11):2464–70; discussion 70–1. Epub 1999/11/05. 10.1161/01.str.30.11.2464 . 10548685

[29] Reglodi D, Somogyvari-Vigh A, Maderdrut JL, Vigh S, Arimura A. Postischemic spontaneous hyperthermia and its effects in middle cerebral artery occlusion in the rat. Experimental neurology. 2000;163(2):399–407. Epub 2000/06/02. 10.1006/exnr.2000.7367 . 10833314

[30] Liu F, Schafer DP, McCullough LD. TTC, fluoro-Jade B and NeuN staining confirm evolving phases of infarction induced by middle cerebral artery occlusion. Journal of neuroscience methods. 2009;179(1):1–8. Epub 2009/01/27. 10.1016/j.jneumeth.2008.12.028 19167427 PMC2674851

[31] Chen ST, Hsu CY, Hogan EL, Maricq H, Balentine JD. A model of focal ischemic stroke in the rat: reproducible extensive cortical infarction. Stroke; a journal of cerebral circulation. 1986;17(4):738–43. 10.1161/01.str.17.4.738 . 2943059

[32] Liu YC, Lee YD, Wang HL, Liao KH, Chen KB, Poon KS, et al Anesthesia-Induced Hypothermia Attenuates Early-Phase Blood-Brain Barrier Disruption but Not Infarct Volume following Cerebral Ischemia. PloS one. 2017;12(1):e0170682 Epub 2017/01/25. 10.1371/journal.pone.0170682 28118390 PMC5261567

[33] Liao KH, Wei VC, Wang HL, Chen HY, Lai TW. Carbogen inhalation opens the blood-brain barrier in rats without causing long-term metabolic or neurological deficit. Brain research. 2019;1720:146320 Epub 2019/07/06. 10.1016/j.brainres.2019.146320 . 31276640

[34] Aarts M, Liu Y, Liu L, Besshoh S, Arundine M, Gurd JW, et al Treatment of ischemic brain damage by perturbing NMDA receptor- PSD-95 protein interactions. Science. 2002;298(5594):846–50. Epub 2002/10/26. 10.1126/science.1072873 . 12399596

[35] Esneault E, Castagne V, Moser P, Bonny C, Bernaudin M. D-JNKi, a peptide inhibitor of c-Jun N-terminal kinase, promotes functional recovery after transient focal cerebral ischemia in rats. Neuroscience. 2008;152(2):308–20. Epub 2008/02/12. 10.1016/j.neuroscience.2007.12.036 . 18262367

[36] Taghibiglou C, Martin HGS, Lai TW, Cho T, Prasad S, Kojic L, et al Role of NMDA receptor-dependent activation of SREBP1 in excitotoxic and ischemic neuronal injuries. Nature medicine. 2009;15(12):1399–U7. 10.1038/nm.2064 WOS:000272407800020. 19966780

[37] Lai TW, Shyu W-C, Wang YT. Stroke intervention pathways: NMDA receptors and beyond. Trends in Molecular Medicine. 2011;17(5):266–75. 10.1016/j.molmed.2010.12.008 WOS:000291294900005. 21310659

[38] Lai TW, Wang YT. Fashioning drugs for stroke. Nature medicine. 2010;16(12):1376–8. 10.1038/nm1210-1376 WOS:000285048900022. 21135846

[39] Hill MD, Martin RH, Mikulis D, Wong JH, Silver FL, Terbrugge KG, et al Safety and efficacy of NA-1 in patients with iatrogenic stroke after endovascular aneurysm repair (ENACT): a phase 2, randomised, double-blind, placebo-controlled trial. Lancet Neurol. 2012;11(11):942–50. Epub 2012/10/12. 10.1016/S1474-4422(12)70225-9 . 23051991

[40] Bonny C, Oberson A, Negri S, Sauser C, Schorderet DF. Cell-permeable peptide inhibitors of JNK: novel blockers of beta-cell death. Diabetes. 2001;50(1):77–82. Epub 2001/01/09. 10.2337/diabetes.50.1.77 . 11147798

[41] Borsello T, Clarke PG, Hirt L, Vercelli A, Repici M, Schorderet DF, et al A peptide inhibitor of c-Jun N-terminal kinase protects against excitotoxicity and cerebral ischemia. Nature medicine. 2003;9(9):1180–6. Epub 2003/08/26. 10.1038/nm911 . 12937412

