# Supplementary material for: Hypothermia but not NMDA receptor antagonism protects against stroke induced by distal middle cerebral arterial occlusion in mice
Source: PLoS One. 2020 Mar 3;15(3):e0229499. doi: 10.1371/journal.pone.0229499 (PMC7053748; doi:10.1371/journal.pone.0229499)
Supplement: S1 Fig — (A) Representative photo of a mouse dMCA imaged following craniectomy. (B) Photo image showing a needle placed under the arterial branch to be occluded by suture ligation. (PDF) [file pone.0229499.s001.pdf]

A B

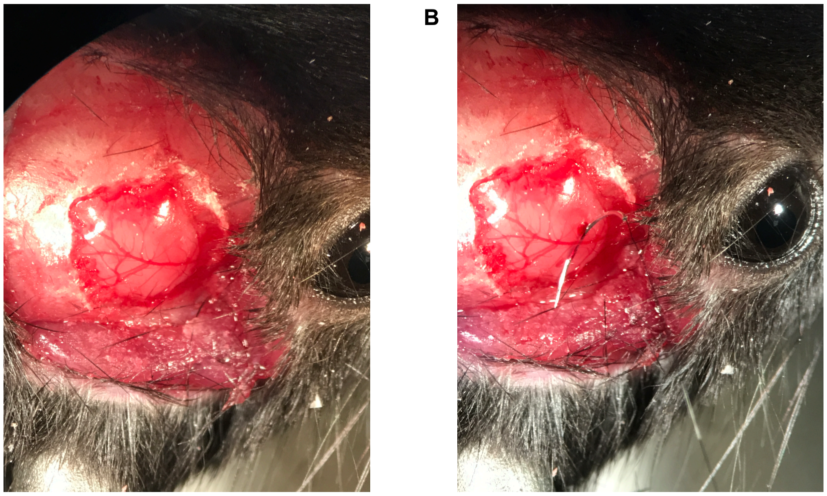

Supplementary Fig 1. Photographic demonstration of the distal middle cerebral artery (dMCA) and its occlusion. (**A**) Representative photo of a mouse dMCA imaged following craniectomy. (**B**) Photo image showing a needle placed under the arterial branch to be occluded by suture ligation.
